# Cytomegalovirus Disease as a Risk Factor for Invasive Fungal Infections in Liver Transplant Recipients under Targeted Antiviral and Antimycotic Prophylaxis

**DOI:** 10.3390/jcm12165198

**Published:** 2023-08-09

**Authors:** Robert Breitkopf, Benedikt Treml, Zoran Bukumiric, Nicole Innerhofer, Margot Fodor, Aleksandra Radovanovic Spurnic, Sasa Rajsic

**Affiliations:** 1 Department of Anesthesia and Intensive Care Medicine, Medical University Innsbruck, 6020 Innsbruck, Austrianicole.innerhofer@i-med.ac.at (N.I.); 2 Institute of Medical Statistics and Informatics, Faculty of Medicine, University of Belgrade, 11000 Belgrade, Serbia; 3 Department of Visceral, Transplantation and Thoracic Surgery, Medical University of Innsbruck, 6020 Innsbruck, Austria; margot.fodor@tirol-kliniken.at; 4 Clinic for Infectious and Tropical Diseases, University Clinical Centre of Serbia, 11000 Belgrade, Serbia

**Keywords:** cytomegalovirus, invasive fungal infections, liver transplantation, serostatus, virus load

## Abstract

Cytomegalovirus (CMV) infection is the most common opportunistic infection that occurs following orthotopic liver transplantation (OLT). In addition to the direct infection-related symptoms, it also triggers an immunological response that may contribute to adverse clinical outcomes. CMV disease has been described as a predictor of invasive fungal infections (IFIs) but its role under an antiviral prophylaxis regimen is unclear. Methods: We retrospectively analyzed the medical records of 214 adult liver transplant recipients (LTRs). Universal antiviral prophylaxis was utilized in recipients with CMV mismatch; intermediate- and low-risk patients received pre-emptive treatment. Results: Six percent of patients developed CMV disease independent of their serostatus. The occurrence of CMV disease was associated with elevated virus load and increased incidence of leucopenia and IFIs. Furthermore, CMV disease was associated with higher one-year mortality and increased relapse rates within the first year of OLT. Conclusions: CMV disease causes significant morbidity and mortality in LTRs, directly affecting transplant outcomes. Due to the increased risk of IFIs, antifungal prophylaxis for CMV disease may be appropriate. Postoperative CMV monitoring should be considered after massive transfusion, even in low-risk serostatus constellations. In case of biliary complications, biliary CMV monitoring may be appropriate in the case of CMV-DNA blood-negative patients.

## 1. Introduction

Liver transplant recipients (LTRs) are at an increased risk of opportunistic infections, particularly cytomegalovirus (CMV) and invasive fungal infections (IFIs), both directly determining graft survival, patient morbidity, and mortality. This holds especially true in donor seropositive/recipient seronegative (D+/R−) high-risk constellations since latent allograft CMV may become activated due to the absence of CMV antibodies. Without prophylactic measures, up to 30% of LTRs may develop CMV disease [1,2].

Attributable symptoms of CMV disease include either viral syndrome with fever, malaise, and/or blood count alterations, or a tissue-invasive disease characterized by hepatitis, pneumonitis, retinitis, or gastrointestinal CMV disease [3].

Furthermore, CMV infections can trigger an indirect immune response, leading to allograft injury or rejection, increasing the risk of graft vasculopathy with secondary lesions (e.g., chronic allograft nephropathy, bronchiolitis obliterans, coronary artery disease, etc.) or superinfection with opportunistic pathogens (e.g., fungal infections) and posttransplant lymphoproliferative disorder (PTLD) [4,5,6,7].

Cytomegalovirus and IFIs share some of the risk factors for occurrence and indirectly indicate the level of ongoing immunosuppression. Despite the known association between CMV and IFIs after stem cell transplantations, data from solid organ transplant recipients is still limited and inconclusive, especially in the context of applied prophylactic measures [8,9].

Prophylactic regimes for CMV vary widely between transplant centers, depending mainly on the donor and recipient serostatuses. High-risk LTR (D+/R−) should receive 3–6 months of CMV prophylaxis, while 3 months of prophylaxis may be sufficient for intermediate-risk seropositive recipients [10]. In low-risk seronegative patients, no antiviral prophylaxis is generally recommended, provided CMV-negative blood or leucodepleted blood products were used. Therefore, commonly used strategies include universal prophylaxis (antiviral therapy for all LTRs) and pre-emptive therapy (virus load monitoring and treatment in the early stages of infection, before symptoms develop) [3]. Finally, pre-emptive therapy has the potential benefit of reducing drug toxicity (e.g., leucopenia, nephrotoxicity, etc.) through more targeted use.

In addition to serostatus, the association between viral load and the development of CMV disease is a subject of ongoing research. Higher viral loads are indicative of the development of CMV disease [11,12] and late CMV disease [13], as well as an increase in non-relapse and overall mortality [14].

We hypothesized that early postoperative CMV disease impairs one-year mortality and may be associated with an increased risk of opportunistic infections, particularly IFIs. Therefore, we aimed to investigate the incidence of CMV disease and to identify its possible risk factors, including serostatus and viral load, under targeted, risk-adjusted antiviral prophylaxis. Moreover, we summarize the direct and indirect effects of early postoperative CMV infections following orthotopic liver transplantation (OLT) and its association with procedural complications, focusing particularly on IFIs and the impact on one-year outcomes.

## 2. Materials and Methods

### 2.1. Patient Selection

We retrospectively reviewed the electronic medical charts of all adult LTRs undergoing first-time OLT of deceased donor graft (time range January 2017–December 2020) at the Medical University of Innsbruck, Austria. Patients under the age of 18 and those needing re-transplantation, combined organ transplantation, or transplantation of living liver donation were excluded. In the case of early re-transplantation (within the study period), only the first operation was analyzed.

The study was approved by the Ethics Committee of the Medical University of Innsbruck, Austria (number 1126/2022). We prepared and revised our work based on the strengthening of the reporting of observational studies in epidemiology (STROBE) statement—a checklist of items (Table S1).

### 2.2. Study Design and Definitions

The aim of our work was to evaluate the risk factors (including serostatus and viral load) and consequences of early CMV disease following OLT. The primary endpoint was the incidence of CMV disease within 90 days of surgery. The secondary endpoints included evaluation of postoperative complications (e.g., IFIs, leucopenia, acute kidney injury, etc.), direct (CMV syndrome, myelosuppression, tissue-invasive disease) and indirect (immunological, vascular, and biliary complications; PTLD) effects of CMV disease within 90 days of OLT, and the one-year outcome regarding CMV relapse, graft survival, and mortality.

We obtained data on (1) sociodemographic characteristics, including CMV serostatus, (2) underlying disease and transplantation indication, (3) the surgical technique, including data on organ donation and preservation, (4) immunosuppression and antimicrobial prophylaxis, (5) postoperative CMV infections (including virus load and the day of occurrence) and proven or probable IFIs, and (6) potential postoperative complications and clinical outcomes.

Invasive fungal infections were diagnosed following the definitions of the European Organization for Research and Treatment of Cancer/Invasive Fungal Infections Cooperative Group and the National Institute of Allergy and Infectious Diseases Mycoses Study Group (EORTC/MSG) Consensus Group [15].

Diagnosis of a CMV infection was made based on confirmation of molecular CMV replication, regardless of symptoms. CMV disease included attributable symptoms of either viral syndrome (temperature ≥ 38 °C, severe malaise, or leucopenia defined as white blood cell (WBC) count of <3500/µL if the WBC prior to the development of clinical symptoms was ≥4000/µL or a decrease of >20% if the WBC count prior to the development of clinical symptoms was <4000/µL) and/or tissue invasive disease, as recommended by the American Society of Transplantation or the CMV Drug Development Forum [16,17].

Cytomegalovirus hepatitis was diagnosed by detecting CMV in a viral culture of liver biopsy tissue, or CMV inclusions by histopathology/cytology, immunohistochemical analysis, or in situ hybridization. The presence of other pathogens or etiologies of hepatic dysfunction such as rejection did not exclude the diagnosis of CMV hepatitis.

Gastrointestinal CMV disease was defined by the detection of CMV (viral culture) in tissue biopsies, histopathology/cytology with CMV inclusions, immunohistochemical analysis, or in situ hybridization in addition to ongoing upper or lower gastrointestinal tract symptoms and/or signs such as nausea, vomiting, anorexia, dysphagia, odynophagia, cramping, diarrhea, or abdominal pain (not excluding the diagnosis in case of the presence of other pathogens).

Considering the high risk of bleeding and pneumothorax in the case of a lung biopsy, the combination of quantitative plasma PCR, high virus load (>5000 IU/mL) in bronchoalveolar lavage, and radiological signs was accepted for CMV pneumonitis diagnosis [18,19,20].

Resistant and refractory CMV infection and disease were defined as recommended by the CMV Resistance Working Group of the CMV Drug Development Forum [21]. Where applicable, sequencing for resistance mutations was performed.

If acute cell rejection (ACR) was suspected, and based on the patient’s clinical condition, an immediate biopsy was initiated. Histological examination of the biopsy specimens was performed by an experienced pathologist and examined for portal vein inflammation, bile duct inflammation damage, and venous endothelial inflammation. All ACRs were classified based on the Banff Rejection Activity Index and further treatment was based upon histological evidence. Mild rejection (rejection activity index ≤ 4) was managed by optimizing baseline immunosuppression, whereas biopsy-proven moderate to severe acute rejection (RAI ≥ 5) received high-dose glucocorticoids (e.g., 500–1000 mg methylprednisolone) for 1–3 days, followed by glucocorticoid taper in addition to optimized maintenance immunosuppression. At our institution, anti-thymocyte globulin (ATG) is the treatment regimen of choice for rare cases of biopsy-proven, glucocorticoid-refractory acute rejection (1.5 mg/kg daily, administered intravenously for 5–7 days).

Diagnosis of antibody-mediated rejection (ABMR) was made based on the recommendation of the Banff working group upon pathological histological findings with proof of C4d staining, screening for donor-specific anti-human leucocyte antigen antibodies (DSA) and excluding other causative factors [22]. We treated mild ABMR in the same way as ACR. In cases of moderate or severe ABMR, we used plasmapheresis and intravenous immunoglobulin with or without B cell-directed therapy, depending on the severity and time course after OLT, as well as the patient’s condition.

### 2.3. Surgical Technique and Immunosuppression

Standard OLT was defined as cadaver transplantation of a standard criteria organ and donation after brain death with subsequent static cold storage. Since February 2018, we have used normothermic machine perfusion for donor or recipient-related indications, as well as for logistic reasons in cases of limited resources. Standard implantation was performed via retrohepatic cava resection without a veno-venous bypass and a duct-to-duct biliary anastomosis.

Standard immunosuppression was given as a triple therapy of a tapered corticosteroid, tacrolimus, at a target C0 level of 7–10 ng/mL, and mycophenolate mofetil without induction of T-cell depletion.

### 2.4. Antimicrobial Prophylaxis and Surveillance

Piperacillin/tazobactam or levofloxacin—in case of β-lactam allergy—was administered as an antibiotic prophylaxis. Trimethoprim/sulfamethoxazole was administered for 6–12 months as a universal pneumocystis prophylaxis, starting as soon as the patient was stable.

Echinocandins were administered as a targeted antimycotic prophylaxis for at least 7–14 days in case of two or more predefined perioperative risk factors (MELD score > 30, serum creatinine > 2 mg/dL, or preexisting end-stage renal disease, known fungal colonization, antibiotic pretreatment up to three months before transplantation, high-urgency indication, split-liver transplantation, intraoperative massive transfusion, prolonged operation duration, Roux-Y-choledochojejunostomy, or known donor-derived infection). Echinocandins are an increasingly used option for IFI prophylaxis [23]. Although most Candida albicans species from the early postoperative period are still susceptible to the currently used fluconazole, echinocandins and amphotericin have the advantage of being active against the increasing number of non-albicans Candida species as well as Aspergillus species. Compared with azoles, echinocandins are also easier to dose, show no pharmacological interactions with the used immunosuppressives, and lack the nephrotoxicity of amphotericin [24,25]. Postoperative initiation of prophylaxis was warranted in cases of acute kidney injury, need for relaparotomy, occurrence of bile leaks, or CMV infections. We used fluconazole, voriconazole, or liposomal amphotericin B in cases of preexisting echinocandin-resistant colonization or donor-derived infection identified in the machine perfusion fluid. Systemic donor infection was considered an absolute contraindication for cadaveric organ donation.

Cytomegalovirus IgG serology test was performed for all donors and recipients as part of the risk stratification before OLT. The recipient was further tested at the time of transplantation if the preoperative serology was negative (R−). For antiviral prophylaxis, we generally followed a pre-emptive regimen with weekly molecular monitoring of low- and intermediate-risk LTRs for three months, except in high-risk D+/R− patients who received a universal prophylaxis of valganciclovir (for 3–6 months) after initial application of intravenous ganciclovir supplemented with CMV-specific hyperimmunoglobulin (100 IE/kg) within the first 72 h following OLT, if immediate attainment of a therapeutic dose was not possible (e.g., due to renal dysfunction, leucopenia, or thrombopenia). In cases of critical illness (including mechanical ventilation or septic shock), intravenous ganciclovir was preferred over valganciclovir.

To avoid transfusion-transmitted CMV, only leucoreduced blood products were transfused.

We used weekly quantitative plasma nucleic acid amplification testing, calibrated to the WHO standard for diagnosis of CMV infection (Cobas^®^ 6800/8800, Roche Diagnostics, Basel, Switzerland), guided pre-emptive strategies, and monitored responses to therapy. The lower limit of detection for this assay (95% reproducibility limit) is 20 IU/mL.

To assess the dose–response relationship, CMV viral load was divided into three categories: (1) 125–500 IU/mL, (2) 501–1000 IU/mL, and (3) more than 1000 IU/mL. The lowest threshold of 125 IU/mL arises from the lower limit of quantification of the assay used; the upper threshold of 1000 IU/mL was chosen based on the earlier cut-off for pre-emptive therapy initiation [26,27]. The pre-emptive antiviral therapy was applied once DNAemia exceeded 125 IU/mL and was continued until CMV DNA was undetectable in two consecutive samples taken within two weeks.

In case of suspected tissue invasion, histopathologic examination, including immunohistochemistry, was performed, provided the intervention risk was acceptable.

Finally, we performed routine microbiological screening (at least once a week) for prospective surveillance of healthcare-associated infections, defined in the ECDC criteria. Antimicrobial susceptibility was tested using the European Committee on Antimicrobial Susceptibility Testing standards [28,29,30]. Positive samples taken via drains more than 24 h in situ, as well as from respiratory secretions, wound sites, skin, stool, and asymptomatic candiduria were not treated but interpreted as colonization. If feasible, the management of colonization was directed at the elimination of predisposing factors.

### 2.5. Statistical Analyses

All statistical analyses were performed using SPSS (Version 22.0. Released 2013, Armonk, NY, USA: IBM Corp.). A significance level of 0.05 was applied, and all statistical assessments were two-sided. Depending on data distribution and the type of variables, we present the results as median (range, minimum–maximum), mean with standard deviation, and frequency (percentage). The Mann–Whitney U test was used for ordinal and nonnormal numeric data, and the independent samples t-test was used for parametric data. Finally, Chi-square and Fisher’s exact tests were used to analyze the differences between nominal data (frequencies).

## 3. Results

### 3.1. Donor, Recipient, and Procedural Characteristics

Out of the 299 adult patients who underwent an OLT during the study period, 85 (28%) were excluded (42 were younger than 18 years, 32 underwent re-transplantation, and 11 had combined organ transplantations). We included 214 LTRs—76% male patients (163/214) and a mean age of 57 ± 11 years—in the final analysis (Table 1).

The main indications for OLT were tumors (42%, 89/214) and cirrhosis (32%, 68/214); acute liver failure was the indication for liver transplantation in 5% (10/214) of the cases.

The majority of accepted grafts were donations after brain death (92%, 196/214), and normothermic machine perfusion was used in 33% (70/214) of the cases. Biliary reconstruction was mainly performed via duct-to-duct anastomosis (93%, 199/214), while split-liver transplantation was performed in 3% (6/214) of patients (Table S2).

Among the analyzed risk factors, only elevated intraoperative blood transfusion was associated with an increased risk of CMV infection (3800 vs. 2257 mL, *p* = 0.011) (Table S2).

Cytomegalovirus seroprevalence in our study population was 48% (103/214), and antiviral prophylaxis was utilized in 28% (59/214) of the patients.

### 3.2. Early-Onset CMV-Infection

#### 3.2.1. Incidence and Clinical Characteristics

Early postoperative CMV infection was detected in 23% (50/214) of patients, with a median time to onset of 23 (2–85) days. CMV occurred as the primary infection in 14% (7/50) of the cases, with a median time to onset of 48 (4–79) days in previously unexposed LTRs; five of these were breakthrough infections in recipients under universal antiviral prophylaxis (D+/R− status). In 86% (43/50) of the cases, CMV emerged as a reactivation of a pre-existing latent infection (median time to onset of 21 (2–85) days, Table S3).

Regarding the serological status, the incidence of CMV infection was 9% (5/59) among D+/R− recipients under ongoing universal prophylaxis, 42% (43/103) among CMV-seropositive (CMV R+) with pre-emptive prophylaxis, and 4% (2/52) among D−/R− patients (Table S3). Most of the infections occurred in the seropositive recipient group (86%, 43/50) and the CMV mismatch group under ongoing prophylaxis (10%, 5/50).

The median CMV load was 552 IU/mL (146–1,057,871). Most patients developed low-level DNAemia of <500 IU/mL (48%, 24/50); 14% (7/50) of the patients had intermediate DNAemia (501–1000 IU/mL), and 38% (19/50) had high-level infections (>1000 IU/mL). Three patients (1%, 3/214) were evaluated as non-infectious due to detected virus loads below the lower limit of quantification (Tables S3 and S4).

#### 3.2.2. Direct Effects

Six percent (13/214) of the patients developed CMV disease with attributable symptoms. The occurrence of CMV disease was associated with significantly higher viral load in comparison to CMV infection (6010 IU/mL; 211–1,057,871 vs. 518 IU/mL; 146–4500, *p* = 0.012) (Table S3).

**Figure 1:**
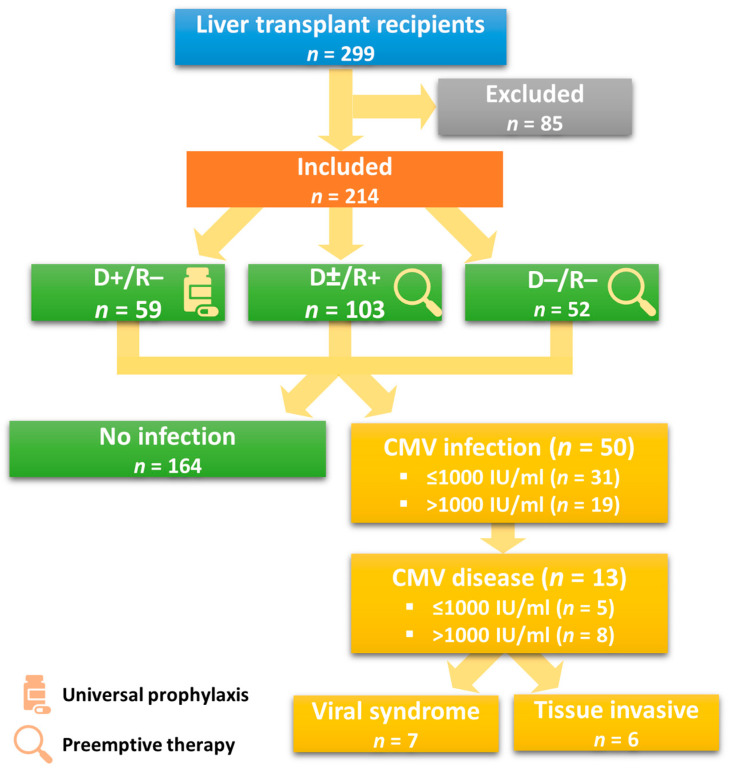
Patient recruitment based on serostatus and type of prophylaxis, including viral load and type of CMV disease (*n* = 299).

CMV disease manifested as a viral syndrome in 54% (7/13) of the patients and as a tissue-invasive disease (equally involving the gastrointestinal tract, transplanted liver allograft, and lungs in the form of pneumonitis) in 46% (6/13) of the patients (Figure 1).

Cytomegalovirus disease was associated with a higher rate of IFIs (39% vs. 10%, *p* = 0.012) and a higher overall mortality rate (46% vs. 11%, *p* = 0.003), with a tendency towards an increased rate of ischemic-type biliary lesion (23% vs. 6%, *p* = 0.058) (see Table 2).

**Figure 2:**
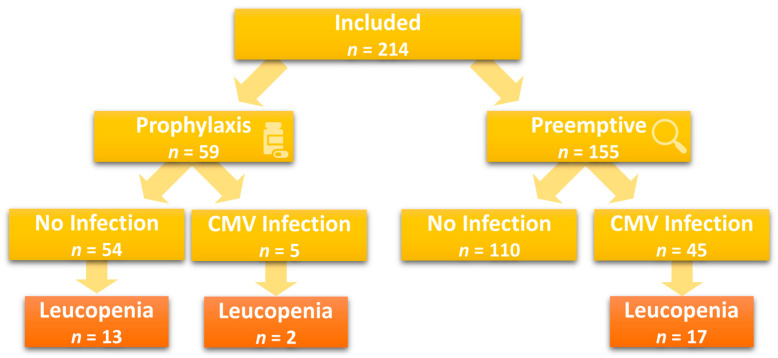
Incidence of leucopenia based on prophylaxis type and occurrence of CMV infection (*n* = 214).

Induced leucopenia occurred in 15% (32/214) of the patients, and more often in association with CMV infection (38% vs. 8%, *p* < 0.001) or CMV disease (50% vs. 14%, *p* = 0.018). In 41% (13/32) of the patients, leucopenia was drug-related, occurring during ongoing antiviral prophylaxis and concomitant use of mycophenolate mofetil without CMV infection. Most of the patients (53%, 17/32) developed de-novo leucopenia at the time of CMV infection, without antiviral prophylaxis. Among the patients under universal prophylaxis, only two (3%, 2/59) developed leucopenia during a breakthrough infection (Figure 2).

### 3.3. Postoperative Complications

The mean length of initial ICU stay was 5 (1–117) days. We observed a direct association between bile leak occurrence and CMV infections (20% vs. 6%, *p* = 0.009), independent of patient serostatus or viral load (Table S5).

Twelve percent (26/214) of the patients developed IFIs; 73% (19/26) of them had *Candida* spp., while 19% (5/26) had *Aspergillus* spp. as the predominant fungal pathogens. Other pathogens such as *Saccharomyces cerevisiae*, *Geotrichum capitatum*, *Fusarium* spp., *Penicillium* spp., and *Mucor circinelloides* were identified as part of a mixed flora. The occurrence of IFIs was independent of CMV infection (16% vs. 11%, *p* = 0.305), serostatus, and viral load but was associated with the occurrence of CMV disease (38% vs. 10%, *p* = 0.012). In the case of CMV disease, we identified more than five times increased risk of IFIs (OR 5.36, 95% CI: 1.61–17.88, *p* = 0.006).

Over the first 90 days following OLT, 44% (94/214) of the patients developed acute kidney failure. There were no significant differences in the occurrence of AKI associated with infection, viral load, or prophylaxis type, i.e., universal prophylaxis vs. pre-emptive therapy.

### 3.4. One-Year Outcome

#### 3.4.1. Mortality

The overall one-year mortality was 14% (29/214) and was significantly higher in patients with CMV disease (46% vs. 11%, *p* = 0.003). CMV infections and viral loads were not associated with mortality. Patients with CMV disease had more than six times higher risk of death within the first year following OLT (OR 6.63, 95% CI: 2.05–21.45, *p* = 0.002). Eight percent (17/214) of the patients died in the context of a graft failure.

The main cause of death was infection (62%, 18/29), of which 61% (11/18) were due to bacterial infections and 39% (7/18) were due to fungal infections. These were followed by procedural complications (14%, 4/29), myocardial infarction (7%, 2/29), cerebrovascular accidents (7%, 2/29), de-novo occurrence of malignancies (PTLD; 7%, 2/29), and respiratory failure due to COVID-19 (3.4%, 1/29).

#### 3.4.2. Graft Survival

Within the first year of OLT, 17 patients (8%, 17/214) developed graft failure within a median of 52 days (1–300 days), independent of serostatus or viral load (Table 2). Two of these patients died before being assigned to a re-transplantation waiting list; the others were re-listed and nine (60%, 9/15) were retransplanted within one year. More than half of these patients (56%, 5/9) survived and five of them (56%) were prioritized for either hepatic artery thrombosis (*n* = 2) or primary non-function (*n* = 3); one patient was transplanted a third time within the first year of the first OLT. Of the six patients remaining on the waiting list, two died and four survived the first year while being actively listed for re-transplantation.

Given the overall mortality of 14% (29/214) and nine (4%, 9/214) re-listed for graft failure, we calculated a death-censored one-year graft survival rate of 95% (176/185).

#### 3.4.3. CMV-Relapse Rate

Independent of the initial viral load, CMV relapse after infection was observed in 36% (18/50) of the patients. Most infections occurred in seropositive recipients (14% vs. 4%, *p* = 0.012), and 62% (8/13) of patients with CMV disease relapsed within one year of OLT, in contrast to 27% (10/37) with CMV infection only. Consequently, the likelihood of a relapse after the disease was more than double in comparison to after infection (OR 2.28, 95% CI: 1.15–4.50, *p* = 0.018). None of the cases met the criteria for resistant or refractory CMV infection or disease.

### 3.5. Indirect Effects of Early-Onset CMV Infection

#### 3.5.1. Immunological Complications

Twenty patients (9%, 20/214) developed ACR, with 6% (12/214) of these occurring late (3–6 months after OLT), at the time of cessation of initial higher immunosuppression. There was a significant association between early-onset rejection and postoperative CMV infection (63% vs. 20%, *p* = 0.020) (see Table S5).

One patient developed progressive cholestatic graft dysfunction due to chronic rejection, while another one developed ABMR. A further patient died due to a cerebrovascular accident during a central nervous system involvement of graft-versus-host disease (Table S5). Finally, we observed a significantly higher risk of overall immunological complications in seropositive compared to seronegative recipients (17% vs. 7%, *p* = 0.027), independent of viral load when the infection was present (see Tables S6 and S7).

#### 3.5.2. Vascular Complications

Vascular complications were observed in 14% (30/214) of the patients: hepatic artery thrombosis (HAT) in 5% (11/214) of the patients, hepatic artery stenosis in 1% (3/214), portal vein thrombosis (PVT) in 5% (10/214), venous thromboembolism (VTE) other than PVT in 2% (4/214), including one patient with pulmonary embolism, and arterial thrombosis other than HAT was observed in 2% (5/214) of the patients, including two patients with myocardial infarction. One patient with PVT, two with HAT, and two with arterial thrombosis suffered preceding CMV infections (all with moderate virus loads), whereas CMV infections were not detected in any of the patients with VTE.

Concerning the outcome of the hepatic artery pathologies for graft survival, eight of the patients with HAT (73%, 8/11) had early onset (within 30 days of OLT), independent of CMV serostatus, infection, and viral load. Patients with concomitant CMV infection had a moderate CMV load; four of them (36%, 4/11) died within one year (three after re-transplantation, one due to hepatic necrosis within the first year of OLT while on the waiting list), two were successfully retransplanted within 14 days, and two were still under evaluation for re-transplantation one year after OLT. Another patient was re-listed because of ischemic cholangiopathy due to hepatic artery stenosis, and two further cases of hepatic stenosis were treated conservatively.

#### 3.5.3. Non-Anastomotic Biliary Strictures (NAS) and Post-Transplant Lymphoproliferative Disorder (PTLD)

Twenty-seven (13%) of the LTRs had non-anastomotic biliary lesions within the first year of OLT, with 7% of the patients (15/214) presenting as ischemic-type biliary lesions due to chronic ductopenic rejection or ischemia-reperfusion injury and 6% (12/214) presenting as intrahepatic biliary lesions due to HAT (*n* = 8), hepatic stenosis (*n* = 3), or severe low-cardiac output (*n* = 1). The patients with ischemic-type biliary lesions showed a trend towards a possible causal relationship with CMV infection (78% vs. 44%, *p* = 0.053). Additionally, seropositivity due to previous exposure to CMV (11% vs. 4%, *p* = 0.059) was observed.

Finally, two patients developed fatal PTLD in the first year of OLT, both after low-level CMV reactivation.

## 4. Discussion

Cytomegalovirus remains the most common opportunistic infection among liver transplant recipients due to its high prevalence (60–90%) as a latent and asymptomatic infection [31,32]. Without prophylaxis, CMV infections usually occur within the first 90 days of OLT [33]. Risk factors for CMV infections include the serological statuses of the donor and recipient, the degree of immunosuppression, and the use of antiviral prophylaxis [34]. CMV infections have been reported with an incidence of up to 88% in high-risk recipients not receiving prophylaxis and 13% in cases with low-risk profiles [35,36].

In the present retrospective analysis of 214 adult first-time LTRs, we confirmed a CMV-infection incidence of 23% in the first 90 days of OLT. The majority of CMV infections were temporal, low-level DNAaemias that were successfully treated. We did not identify any case with resistant or refractory CMV infection, consistent with published evidence, with an incidence of less than 5% in solid organ transplant recipients [37,38,39,40]. Given the mean onset time of postoperative CMV DNAemia and the time criterion for the diagnosis, we assumed only moderate relevance of resistant/refractory CMV infections for the early postoperative phase after first-time OLT. However, clinicians should be aware of the change in risk when newer antiviral drugs with lower resistance barriers are used (e.g., letermovir and maribavir) [38,41,42,43,44].

Given a preoperative CMV seroprevalence of 48% among the LTRs, no difference was observed in the time of onset between patients under universal prophylaxis and those undergoing pre-emptive therapy.

Almost all the identified cases (86%) manifested as reactivations, and less than 1% developed de-novo infections, i.e., were not transmitted during the transplantation. The analysis of patient characteristics and underlying disease revealed a homogenous distribution between the CMV and non-CMV groups. Of the procedural characteristics, only elevated intraoperative blood transfusion was associated with increased risk of CMV infection despite the use of leucodepleted blood products (*p* = 0.011), which is of clinical interest, keeping the mentioned de-novo infections in mind. As previously observed by Strauss et al., a transfusion-transmitted CMV infection cannot be completely prevented by leucocyte depletion, especially during the early onset of primary infection in the blood donor. At that time, before anti-CMV antibodies are formed, free CMV virions are present in the plasma. Clearance of these occurs following the emergence of CMV antibodies, and the virus is partitioned within leucocytes and can be removed along with the carrier. On the other hand, leucocyte reduction cannot remove free plasma virions [45].

We found an incidence of 6% for CMV disease within the first 90 days of OLT, independent of the prophylactic strategy (universal or pre-emptive) used. This rather low incidence compared with studies describing rates of up to 29% may be associated with the reduced seroprevalence of 48% in the examined study group [3,46,47]. The occurrence of CMV disease was associated with significantly increased viral load. In about half of the patients, CMV disease manifested either as a viral syndrome or tissue-invasive disease. We confirmed earlier findings, as the most often affected organs were the gastrointestinal tract [48] and the transplanted allograft in the form of CMV hepatitis, likely secondary to an abnormal allograft immune response [3,49]. In half the cases of CMV hepatitis, there was suspicion that CMV allograft invasion was donor-transmitted [33,50]. Furthermore, we found an association between reactivation and CMV disease with increased risk of CMV relapse in the first year after OLT.

We proved elevated one-year mortality and morbidity rates of CMV disease, including opportunistic infections (i.e., IFIs) and leucopenia. Given a 90-day IFI-incidence of 12% and despite targeted antimycotic prophylaxis for high-risk LTRs, we found more than five-fold increased risk of IFIs in the case of CMV disease, being associated with increased incidence of leucopenia [24]. The adverse impact of IFIs on postoperative mortality following OLT has recently been investigated, with IFI being identified as a potentially modifiable risk factor in the OLT setting [25,51,52].

In addition to CMV infection/disease, leucopenia can also be associated with the use of myelotoxic antiviral therapy. As already mentioned, all patients received mycophenolic acid for immunosuppressive therapy and *Pneumocystis jirovecii* prophylaxis using trimethoprim/sulfamethoxazole was not started in the mentioned cases of leucopenia. Mild episodes were mostly managed by reduction or discontinuation of the potential causative agent (e.g., valganciclovir, mycophenolic acid, or trimethoprim/sulfamethoxazole). Moreover, persistent or severe episodes were preferably treated using granulocyte colony-stimulating factor since Jorgenson et al. observed that discontinuation of ongoing antiviral therapy might increase the risk of CMV infection, especially in high-risk serological constellations [53,54,55]. Reducing the dose below the recommendation can also increase the risk of antiviral drug resistance [21,56]. With respect to discontinuation and dose reduction of the immunosuppressant (i.e., mycophenolic acid), several studies of kidney transplant recipients reported an increased risk of acute rejection and even graft loss [54,57,58,59,60].

Finally, we observed a significant association between the occurrence of CMV infection with early-onset ACR and postoperative bile leaks, as well as a trend towards increased ischemic-type biliary lesion rate in the first postoperative year. Biliary complications such as leaks or strictures are commonly observed after OLT, with an estimated incidence of up to 32% [61]. Their occurrence is typically time-dependent, with leaks classically seen in the early postoperative period and strictures in the first year after the operation. Based on their site of manifestation, anastomotic lesions are distinguished from non-anastomotic complications of the donor biliary system [62]. Among factors such as hepatic artery stenosis or grafts from donation after cardiac death, postoperative CMV infections have been identified as a significant risk factor for non-anastomotic stricture development [63]. Therefore, our findings might be interpreted in the context of the earlier-described phenomenon of occult CMV infection in the biliary tract, which may obscure the impact of CMV infection on biliary disease following OLT [64,65,66].

### 4.1. Future Perspectives and Outlook

Since the effective use of valganciclovir is associated with a non-negligible risk of myelotoxicity, which is further associated with increased risk of infection, rejection, or development of antiviral resistance, the question of the ideal CMV prophylaxis remains a matter of concern. Current guidelines recommend high-dose valganciclovir (900 mg/day adjusted for renal function) for prophylaxis [3,10]. Khan et al. demonstrated the use of low-dose valganciclovir (450 mg/day, adjusted for renal function) as a safe and effective option for the prevention of CMV disease, with no difference in leucopenia occurrence [67].

Hence, novel antiviral agents like letermovir—a terminase inhibitor with a favorable pharmacokinetic and tolerable profile, particularly without associated myelotoxicity— may be considered valuable alternatives. However, caution is advised since drug-resistant CMV infections (UL56 C325Y resistance mutation) have emerged during its use [68]. Furthermore, relevant drug–drug interactions with immunosuppressants (e.g., cyclosporine-mediated inhibition of OATP1B1/1B3 transporters) must be considered [69].

Addressing the risk of transfusion-transmitted CMV infection, future research on blood-donor serostatus (divided into continuously seronegative, seronegative with subsequent seroconversion, newly seropositive, and long-term seropositive) and quantitative CMV DNAemia in the stored donor plasma is warranted. This could further minimize the risk to solid organ recipients or at least help to estimate the likelihood of transfusion-transmitted versus community-acquired infections [70]. Moreover, occult biliary CMV infection may play an underestimated role in non-anastomotic cholestatic complications following OLT. Chronic CMV latency in bile epithelial cells and virus shedding can trigger chronic inflammation and result in biliary fibrosis. As CMV screening is based on symptomatic DNAemia, the detection of occult biliary CMV infection may have been missed. Along with known factors such as arterial complications, donor age, and cold ischemia time, the possibility of occult CMV infection should be considered as another contributory factor in the development of biliary complications, especially in the case of seropositive recipients [71].

As the current way of administering antiviral prophylaxis is limited by the cost of treatment, effectivity, associated side effects and prophylactic duration, intensive research has been directed towards vaccine development. The vaccine is based on vectored CMV genes, specifically glycoprotein-B and tegument phosphoprotein 65. The immunogenicity and safety of this vaccine have been proven in isolated cases [72,73,74,75,76,77,78,79,80].

### 4.2. Limitations

The present study has several limitations. Due to its retrospective nature, selection bias cannot be excluded. Future prospective research should account for unknown confounders that may have influenced our findings. Moreover, all patients admitted over four consecutive years were included in the analysis, which should minimize the effects of bias. The sample size of the analyzed population is limited by the scope of its monocentric design. Moreover, patients who underwent elective re-transplantation were excluded from the analysis since the inclusion of this very dynamic patient group, which often has previous multifactorial therapies and presentations (e.g., rejection therapy, antimicrobial therapy, multiple hospital admissions, etc.), would affect the analysis due to expressed heterogeneity. The impact of CMV disease on mortality may be limited by possible effects of underlying diseases and comorbidities. The incidence of delayed-onset disease beyond day 90 was not investigated, and the serostatuses of the transfused blood products were not available. Due to the cautious indication for invasive pulmonary biopsy, the frequency of CMV pneumonia may be underrepresented. Likewise, the development of IFI in relation to CMV may be underestimated as it is considered a risk factor for antifungal prophylaxis. Evaluation of the safety and efficacy profiles of drug-related side effects or further anti-herpes benefits of the antiviral prophylaxis used was not the subject of the present study. Moreover, the leucopenia rate might be underestimated, considering a 22% incidence of drug-induced leucopenia in the high-risk non-infection group. However, the data were collected unaffectedly under a uniform treatment regimen.

## 5. Conclusions

Despite prophylactic therapies, the direct and indirect effects of postoperative CMV disease still impact OLT outcomes. We found an association between CMV disease occurrence and increased incidence of IFI, concluding that antifungal prophylaxis should be initiated at the time of CMV disease occurrence. Due to the unlikely but not completely excluded possibility of transfusion-transmitted CMV infections, postoperative CMV PCR monitoring should be considered even in D−/R− LTRs, in case of massive intraoperative transfusion. Moreover, bile CMV monitoring might be appropriate in CMV-DNA blood-negative patients with biliary complications. The development of vaccines and novel drugs for resistant cases is an anticipated future treatment option.

## Figures and Tables

**Table 1:** Demographic and clinical characteristics of the analyzed population (*n* = 214).

Patient Characteristics		All Patients (*n* = 214)	No CMV Infection (*n* = 164)	CMV Infection (*n* = 50)	*p*-Value	Missing (*n*/Total)
Age (years)		57.3 ± 11.1	57.9 ± 10.6	55.5 ± 12.8	0.189	0/214
Male sex		163 (76.2)	127 (77.4)	36 (72.0)	0.451	0/214
Height (cm)		174.2 ± 8.5	174.5 ± 8.2	173.3 ± 9.4	0.405	0/214
Weight (kg)		81.4 ± 16.3	82.4 ± 16.0	78.4 ± 17.2	0.131	0/214
Body mass index (kg/m^2^)		26.8 ± 5.0	27.0 ± 5.0	26.0 ± 4.7	0.182	0/214
SAPS III score		44.8 ± 8.5	44.9 ± 8.5	44.7 ± 8.7	0.897	6/214
MELD score		13 (6–40)	13 (6–40)	13 (6–40)	0.525	10/214
Charlson comorbidity index		4 (0–10)	4 (0–10)	4 (0–8)	0.968	2/214
ICU length of stay (days)		5 (1–117)	5 (1–117)	5.5 (3–40)	0.425	0/214
Invasive fungal infection		26 (12.1)	18 (11.0)	8 (16.0)	0.332	0/214
**Underlying Disease**					0.855	0/214
Acute liver failure		10 (4.7)	7 (4.3)	3 (6.0)	0.702	0/214
Tumors		89 (41.6)	68 (41.5)	21 (42.0)	1.000	0/214
	Hepatocellular carcinoma	82 (92.1)	62 (37.8)	20 (40.0)	0.868	0/214
	Cholangiocellular carcinoma	3 (3.4)	2 (1.2)	1 (2.0)	0.552	0/214
	Neuroendocrine tumor	3 (3.4)	3 (1.8)	0 (0)	1.000	0/214
	Polycystic liver disease	1 (1.1)	1 (0.6)	0 (0)	1.000	0/214
Cirrhosis		68 (31.8)	52 (31.7)	16 (32.0)	1.000	0/214
	Alcoholic cirrhosis	51 (23.8)	41 (25.0)	10 (20.0)	0.571	0/214
	Virus related cirrhosis	9 (4.2)	6 (3.7)	3 (6.0)	0.439	0/214
	Autoimmune cirrhosis	8 (3.7)	5 (3.0)	3 (6.0)	0.393	0/214
Cholestatic disease		15 (7.0)	12 (7.3)	3 (6.0)	1.000	0/214
Nonalcoholic steatohepatitis		14 (6.5)	12 (7.3)	2 (4.0)	0.529	0/214
Metabolic disease		10 (4.7)	7 (4.3)	3 (6.0)	0.702	0/214
Budd-Chiari syndrome		6 (2.8)	5 (3.0)	1 (2.0)	1.000	0/214
Other		2 (0.9)	1 (0.6)	1 (2.0)	0.414	0/214

Abbreviations: SAPS III: simplified acute physiology score III; MELD: model of end-stage liver disease.

**Table 2:** Postoperative complications, one-year outcomes, and indirect effects of CMV disease (*n* = 214).

		All	No Disease	CMV Disease		Missing
Characteristics		(*n* = 214)	(*n* = 201)	(*n* = 13)	*p*-Value	(*n*/Total)
Primary non-function		2 (1.1)	2 (1.0)	0 (0)	1.000	0/214
Early allograft dysfunction		56 (30.9)	50 (24.9)	6 (46.2)	0.107	0/214
Reoperation		81 (37.9)	74 (36.8)	7 (53.9)	0.247	0/214
Bile leak		20 (10.8)	17 (8.5)	3 (23.1)	0.109	0/214
Acute kidney injury		94 (43.9)	87 (43.3)	7 (53.9)	0.567	0/214
Invasive fungal infection		26 (12.1)	21 (10.4)	5 (38.5)	0.012	0/214
	Candidiasis	19 (8.9)	15 (7.5)	4 (30.8)	0.018	
	Aspergillosis	5 (2.3)	4 (2.0)	1 (7.7)		0/214
	Other	2 (0.9)	2 (1.0)	0 (0)		
Overall mortality		29 (13.6)	23 (11.4)	6 (46.2)	0.003	0/214
	Time to death	48 (1–340)	57 (1–340)	44 (26–242)	0.733	0/214
Graft failure		17 (7.9)	16 (8.0)	1 (7.7)	1.000	0/214
	Time to onset	52 (1–300)	35 (1–300)	52 (52–52)	1.000	0/214
Re-transplantation		9 (4.2)	8 (4.0)	1 (7.7)	0.437	0/214
Immunological complications		23 (10.7)	20 (10.0)	3 (23.1)	0.167	9/214
	Acute cellular rejection (early-onset)	8 (34.8)	6 (30.0)	2 (66.7)	0.084	9/214
	Acute cellular rejection (late-onset)	12 (52.2)	12 (60.0)	0 (0)	1.000	9/214
	Chronic rejection	1 (4.3)	1 (5.0)	0 (0)	1.000	9/214
	Antibody-mediated rejection	1 (4.3)	1(5.0)	0 (0)	1.000	9/214
	Graft-versus-host disease	1 (4.3)	0 (0)	1 (33.3)	0.065	9/214
Vascular complications		30 (14.0)	29 (14.7)	1 (7.7)	1.000	9/214
	HAT (early-onset)	8 (26.7)	8 (27.6)	0 (0)	1.000	9/214
	HAT (late-onset)	3 (10.0)	3 (10.3)	0 (0)	1.000	9/214
	Portal vein thrombosis	10 (33.3)	10 (34.5)	0 (0)	1.000	9/214
	Venous thromboembolism	4 (13.3)	4 (13.8)	0 (0)	1.000	9/214
	Arterial thrombotic disease	5 (16.7)	4 (13.8)	1 (100.0)	0.282	9/214
Non-anastomic biliary strictures		27 (12.6)	24 (11.9)	3 (23.1)	0.386	9/214
	Ischemic-type biliary lesion	15 (7.0)	12 (50.0)	3 (100.0)	0.058	9/214
	Intrahepatic biliary lesions	12 (5.6)	12 (50.0)	0 (0)	1.000	9/214
Post-transplant lymphoproliferative disorder		2 (0.9)	1 (0.5)	1 (7.7)	0.123	9/214

Abbreviations: CMV: cytomegalovirus; HAT: hepatic artery thrombosis.

## Data Availability

The datasets used and analyzed in the current study are available from the corresponding author on reasonable request.

## Supplementary Materials

The following supporting information can be downloaded at: https://www.mdpi.com/article/10.3390/jcm12165198/s1, Table S1. STROBE Statement - Checklist of items that should be included in reports of cohort studies; Table S2. Procedural data on transplantation, organ donation and preservation (*n* = 214); Table S3. Postoperative complications and manifestation of CMV infection related to serostatus (*n* = 214); Table S4. Postoperative complications and manifestation of CMV infection related to virus load (IU/mL; *n* = 50); Table S5. Postoperative complications, one-year outcome and indirect effects of CMV infection (*n* = 214); Table S6. One-year outcome and indirect effects of CMV infection related to serostatus (*n* = 214); Table S7. One-year outcome and indirect effects of CMV infection related to virus load (IU/mL; *n* = 50).
