# Supplementary material for: Cytomegalovirus Disease as a Risk Factor for Invasive Fungal Infections in Liver Transplant Recipients under Targeted Antiviral and Antimycotic Prophylaxis
Source: J Clin Med. 2023 Aug 9;12(16):5198. doi: 10.3390/jcm12165198 (PMC10455861; doi:10.3390/jcm12165198)
Supplement: Supplementary file 1 [file jcm-12-05198-s001.zip › jcm-2499746-supplementary.pdf]

Article

CMV Disease as Risk Factor for Invasive Fungal Infections in Liver Transplant Recipients under Targeted Antiviral and Antimycotic Prophylaxis

Supplementary material

Contents:

Table S1. STROBE Statement - Checklist of items that should be included in reports of cohort studies
Table S2. Procedural data on transplantation, organ donation and preservation (n = 214).
Table S3. Postoperative complications and manifestation of CMV infection related to serostatus (n = 214).
Table S4. Postoperative complications and manifestation of CMV infection related to virus load (IU/ml; n = 50).
Table S5. Postoperative complications, one-year outcome and indirect effects of CMV infection (n=214).
Table S6. One-year outcome and indirect effects of CMV infection related to serostatus (n=214).
Table S7. One-year outcome and indirect effects of CMV infection related to virus load (IU/ml; n=50).

Table S1. STROBE Statement - Checklist of items that should be included in reports of cohort studies.

No.	Item	Recommendation	Page
	**Title and abstract**		
1		(*a*) Indicate the study's design with a commonly used term in the title or the abstract	1
		(*b*) Provide in the abstract an informative and balanced summary of what was done and what was found	1
	**Introduction**		
2	Background/rationale	Explain the scientific background and rationale for the investigation being reported	1-2
3	Objectives	State specific objectives, including any prespecified hypotheses	2
	**Methods**		
4	Study design	Present key elements of study design early in the paper	2-5
5	Setting	Describe the setting, locations, and relevant dates, including periods of recruitment, exposure, follow-up, and data collection	2-5
6		(*a*) Give the eligibility criteria, and the sources and methods of selection of participants. Describe methods of follow-up	2-5
	Participants	(*b*) For matched studies, give matching criteria and number of exposed and unexposed	2-5
7	Variables	Clearly define all outcomes, exposures, predictors, potential confounders, and effect modifiers. Give diagnostic criteria, if applicable	2-5
8*	Data sources/ measurement	For each variable of interest, give sources of data and details of methods of assessment (measurement). Describe comparability of assessment methods if there is more than one group	2-5
9	Bias	Describe any efforts to address potential sources of bias	2-5
10	Study size	Explain how the study size was arrived at	-
11	Quantitative variables	Explain how quantitative variables were handled in the analyses. If applicable, describe which groupings were chosen and why	-
12		(*a*) Describe all statistical methods, including those used to control for confounding	5
		(*b*) Describe any methods used to examine subgroups and interactions	5
		(*c*) Explain how missing data were addressed	-
		(*d*) If applicable, explain how loss to follow-up was addressed	-
	Statistical methods	(*e*) Describe any sensitivity analyses	-
	**Results**		
13*		(a) Report numbers of individuals at each stage of study—eg numbers potentially eligible, examined for eligibility, confirmed eligible, included in the study, completing follow-up, and analysed	5
		(b) Give reasons for non-participation at each stage	5
	Participants	(c) Consider use of a flow diagram	7

14*		(a) Give characteristics of study participants (eg demographic, clinical, social) and information on exposures and potential confounders	5-9
		(b) Indicate number of participants with missing data for each variable of interest	-
	Descriptive data	(c) Summarise follow-up time (eg, average and total amount)	5-10
15*	Outcome data	Report numbers of outcome events or summary measures over time	5-10
16		(*a*) Give unadjusted estimates and, if applicable, confounder-adjusted estimates and their precision (eg, 95% confidence interval). Make clear which confounders were adjusted for and why they were included	5-10
		(*b*) Report category boundaries when continuous variables were categorized	5-10
	Main results	(*c*) If relevant, consider translating estimates of relative risk into absolute risk for a meaningful time period	
17	Other analyses	Report other analyses done—eg analyses of subgroups and interactions, and sensitivity analyses	5-10
	**Discussion**		
18	Key results	Summarise key results with reference to study objectives	11
19	Limitations	Discuss limitations of the study, taking into account sources of potential bias or imprecision. Discuss both direction and magnitude of any potential bias	13
20	Interpretation	Give a cautious overall interpretation of results considering objectives, limitations, multiplicity of analyses, results from similar studies, and other relevant evidence	13
21	Generalisability	Discuss the generalisability (external validity) of the study results	13
	**Other information**		
22	Funding	Give the source of funding and the role of the funders for the present study and, if applicable, for the original study on which the present article is based	13

*Give information separately for exposed and unexposed groups.

Table S2. Procedural data on transplantation, organ donation and preservation (n = 214).

**Operative characteristics**	**All patients (n = 214)**	**No CMV infection (n = 164)**	**CMV infection (n = 50)**	**P-value**	**Missing (n/Total)**
Operation duration (minutes)	355 (173-783)	353 (173-783)	362 (188-614)	0.760	0/214
Cold ischemia time (minutes)	435 (125-1199)	433 (130-1061)	471 (125-1199)	0.181	0/214
Intraopertative blood transfusion (ml)	2412 (0-32740)	2257 (0-32740)	3800 (0-11680)	0.011	0/214
Type of graft					0/214
Whole liver	208 (97.2)	160 (97.6)	48 (96.0)	0.626	
Split liver	6 (2.8)	4 (2.4)	2 (4.0)		
Type of biliary anastomosis					0/214
Duct-to-duct	199 (93.0)	153 (93.3)	46 (92.2)	0.755	
Roux-y-choledochojejunostomy	15 (7.0)	11 (6.7)	4 (8.0)		
Type of venous anstomosis					0/214
Retrocaval resection	205 (97.2)	157 (96.9)	48 (98.0)	1.000	
Piggyback	6 (2.8)	5 (3.1)	1 (2.0)		
**Donation and preservation characterstics**					
Type of donation					0/214
Standard criteria donation	46 (21.5)	35 (21.3)	11 (22.0)	1.000	
Extended criteria donation	168 (78.5)	129 (78.7)	39 (78.0)		
Type of donor death					0/214
DBD	196 (91.6)	149 (90.9)	47 (94.0)	0.575	
DCD	18 (8.4)	15 (9.1)	3 (6.0)		
Preservation					0/214
Static cold storage	144 (67.3)	106 (64.6)	38 (76.0)	0.169	
Normothermic machine perfusion	70 (32.7)	58 (35.4)	12 (24.0)		

Intraoperative blood transfusion includes packed red blood cells and autotransfusion of intraoperatively salvaged blood. Abbreviations: SAPS III: simplified acute physiology score III; MELD: model of end stage liver disease; DBD: donation after brain death; DCD: donation after circulatory determination of death.

Table S3. Postoperative complications and manifestation of CMV infection related to serostatus (n = 214).

Characteristics	All (*n* = 214)	*D+/R-* (*n* = 59)	*R+* (*n* = 103)	*D-/R-* (*n* = 52)	P-value	Missing (*n*/Total)
Length of ICU stay (days)	5 (1-117)	5 (1-111)	5 (3-40)	5 (2-117)	0.749	1/214
Acute kidney injury	94 (43.9)	26 (44.1)	46 (44.7)	19 (36.5)	0.400	0/214
Primary non-function	2 (1.1)	0 (0)	2 (1.9)	0 (0)	0.498	0/214
Early allograft dysfunction	56 (30.9)	13 (22.0)	29 (28.2)	14 (26.9)	0.742	0/214
Reoperation	81 ()	19 (32.2)	41 (39.8)	21 (40.4)	0.592	0/214
Bile leak	20 (10.8)	4 (6.8)	8 (7.8)	8 (15.4)	0.247	0/214
Invasive fungal infection	26 (12.1)	8 (13.6)	11 (10.7)	7 (13.5)	0.860	0/214
Candidiasis	19 (8.9)	5 (8.5))	9 (8.7)	5 (9.6)		
Aspergillosis	5 (2.3)	1 (1.7)	2 (1.9)	2 (3.8)	0.579	
Other	2 (0.9)	2 (3.4)	0 (0)	0 (0)		
CMV infection	50 (23.4)	5 (8.5)	43 (41.7)	2 (3.8)	<0.001	0/214
Time to onset (days)	23 (2-85)	50 (4-79)	21 (2-85)	29 (10-48)	0.229	0/50
Virus load	552 (146-1,057,871)	1,032 (149-1,057,871)	518 (146-100,080)	3,300 (591-6,010)	0.512	0/50
CMV disease	13 (6.1%)	2 (3.4)	10 (9.7)	1 (1.9)	0.127	0/214
Viral syndrome	7 (3.3)	1 (1.7)	6 (5.8)	0 (0)	0.706	0/13
Tissue invasive	6 (2.8)	1 (1.7)	4 (3.9)	1 (1.9)		
Virus load	6,010 (211-1,057,871)	540,511 (23,150-1,057,871)	1,9445 (211-100,080)	6,010	0.343	0/13

Abbreviations: CMV: cytomegalovirus; ICU: intensive care unit; D+/R-: donor seropositive/recipient seronegative; R+: recipient seropositive; D-/R-: donor seronegative/recipient seronegative.

Table S4. Postoperative complications and manifestation of CMV infection related to virus load (IU/ml; *n* = 50).

Characteristics	All (*n* = 50)	*>125-500* (*n* =24)	*501-1000* (*n* = 7)	*>1000* (*n* = 19)	P-value	Missing (*n*/Total)
CMV disease	13 (6.1%)	5 (38.5)	0 (0)	8 (61.5)	0.059	0/50
Viral syndrome	7 (53.8)	3 (60.0)	0 (0)	4 (50.0)	1.000	0/13
Tissue invasive	6 (46.2)	2 (40.0)	0 (0)	4 (50.0)		
Postoperative characteristics						
Time to onset (days)	23 (2-85)	20 (2-79)	27 (5-52)	24 (3-85)	0.606	0/50
Length of ICU stay (days)	5.5 (3-40)	6 (3-30)	5.5 (3-15)	5 (3-40)	0.863	0/50
Primary non-function	1 (2.0)	1 (4.2)	0 (0)	0 (0)	1.000	0/50
Early allograft dysfunction	18 (36.0)	8 (33.3)	2 (28.6)	8 (42.1)	0.733	0/50
Reoperation	22 (44.0)	14 (58.3)	3 (42.9)	5 (26.3)	0.068	0/50
Bile leak	10 (20.0)	6 (25.0)	2 (28.6)	2 (10.5)	0.503	0/50
Acute kidney injury	22 (44.0)	13 (54.2)	2 (28.6)	7 (36.8)	0.238	0/50
Invasive fungal infection	8 (16.0)	4 (16.7)	1 (14.3)	3 (15.8)	1.000	0/50
Candidiasis	6 (12.0))	2 (8.3)	1 (14.3)	3 (15.8)		
Aspergillosis	2 (4.0)	2 (8.3)	0 (0)	0 (0)	0.742	0/50
Other	0 (0)	0 (0)	0 (0)	0 (0)		

Abbreviations: CMV: cytomegalovirus; ICU: intensive care unit.

Table S5. Postoperative complications, one-year outcome and indirect effects of CMV infection (n=214).

Characteristics	All patients (*n* = 214)	No Infection (n = 164)	Infection (*n* = 50)	P-value	Missing (*n*/Total)
Primary non-function	2 (1.1)	1 (0.6)	1 (2.0)	0.414	0/214
Early allograft dysfunction	56 (30.9)	38 (23.2)	18 (36.0)	0.097	0/214
Reoperation	81 (37.9)	59 (36.0)	22 (44.0)	0.322	0/214
Bile leak	20(10.8)	10 (6.1)	10 (20.0)	0.009	0/214
Acute kidney injury	94 (43.9)	72 (43.9)	22 (44,0)	1.000	0/214
Invasive fungal infection	26 (12.1)	18 (11.0)	8 (16.0)	0.332	0/214
Candidiasis	19 (8.9)	13 (7.9)	6 (12.0)		0/214
Aspergillosis	5 (2.3)	3 (1.8)	2 (4.0)	0.490	0/214
Other	2 (0.9)	2 (1.2)	0 (0)		0/214
Overall mortality	29 (13.6)	18 (11.0)	11 (22.0)	0.059	0/214
Time to death (days)	49 (1-341)	42 (1-330)	58 (15-341)	0.192	0/29
Graft failure	17 (7.9)	12 (7.3)	5 (10.0)	0.554	0/214
Time to onset (days)	52 (1-300)	59 (1-300)	9 (1-75)	0.154	0/17
Re-transplantation	9 (4.2)	6 (3.7)	3 (6.0)	0.439	0/214
Immunological complications	23 (10.7)	15 (9.1)	8 (16.0)	0.201	9/214
ACR, early-onset	8 (3.7)	3 (1.8)	5 (10.0)	0.020	9/214
ACR, late-onset	12 (5.6)	10 (6.0)	2 (4.0)	0.736	13/214
Chronic rejection	1 (0.5)	1 (0.6)	0 (0)	1.000	13./214
Antibody mediated rejection	1 (0.5)	1 (0.6)	0 (0)	1.000	13/214
Graft-versus-host disease	1 (0.5)	0 (0)	1 (2.0)	0.234	13/214
Vascular complications	30 (14.0)	23 (14.0)	7 (14.0)	0.817	9/214
HAT, early-onset	8 (3.7)	5 (3.0)	3 (6.0)	0.400	9/214
HAT, late-onset	3 (1.4)	3 (1.8)	0 (0)	1.000	9/214
Portal vein thrombosis	10 (4.7)	8 (4.9)	2 (4.0)	1.000	9/214
Venous thromboembolism	4 (1.9)	4 (2.4)	0 (0)	0.574	9/214
Arterial thrombotic disease	5 (2.3)	3 (1.8)	2 (4.0)	0.595	9/214
Non-anastomic biliary strictures	27 (12.6)	18 (11.0)	9 (18.0)	0.230	9/214
Ischemic type biliary lesion	15 (7.0)	8 (4.9)	7 (14.0)	0.053	9./214
Intraheptaic biliary lesions	12 (5.6)	10 (6.0)	2 (4.0)	0.735	9./214
PTLD	2 (0.9)	0 (0)	2 (4.0)	0.056	9/214

Abbreviations: CMV: cytomegalovirus; ACR: acute cellular rejection; HAT: hepatic artery thrombosis; VTE: venous thromboembolism; PTLD: post-transplant lymphoproliferative disorder.

Table S6. One-year outcome and indirect effects of CMV infection related to serostatus (n=214).

	**All (*n* = 214)**	***D+/R-* (*n* = 59)**	***R+* (*n* = 103)**	***D-/R-* (*n* = 52)**	**P-value**	**Missing (*n*/Total)**
Overall mortality	29 (13.6)	4 (6.8)	16 (15.5)	9 (17.3)	0.206	0/214
Time of death (days)	49 (1-341)	145 (1-330)	49 (3-341)	44 (2-279)	0.570	0/29
Graft failure	17 (7.9)	2 (3.4)	11 (10.7)	4 (7.7)	0.290	0/214
Time of onset (days)	52 (1-300)	59 (56-62)	52 (1-300)	7 (1-120)	0.482	0/17
Retransplantation	9 (4.2)	2 (3.4)	5 (4.9)	2 (3.8)	0.913	0/214
Immunological complications	23 (10.7)	4 (6.8)	16 (15.5)	3 (5.8)	0.074	9/214
ACR, early-onset	8 (3.7)	1 (1.7)	5 (4.9)	2 (3.8)	0.586	
ACR, late-onset	12 (5.6)	3 (5.0)	8 (7.8)	1 (1.9)	0.345	
Chronic rejection	1 (0.5)	0 (0)	1 (1.0)	0 (0)	1.000	
Antibody mediated rejection	1 (0.5)	0 (0)	1 (1.0)	0 (0)	1.000	
Graft-versus-host disease	1 (0.5)	0 (0)	1 (1.0)	0 (0)	1.000	
Vascular complications	30 (14.0)	11 (18.6)	12 (11.7)	7 (13.5)	0.546	9/214
HAT, early-onset	8 (3.7)	2 (3.4)	4 (3.9)	2 (3.8)	1.000	
HAT, late-onset	3 (1.4)	2 (3.4)	1 (1.0)	0 (0)	0.445	
Portal vein thrombosis	10 (4.7)	5 (8.5)	2 (1.9)	3 (5.8)	0.157	
Venous thromboembolism	4 (1.9)	1 (1.7)	2 (1.9)	1 (1.9)	1.000	
Arterial thrombotic disease	5 (2.3)	1 (1.7)	3 (2.9)	1 (1.9)	1.000	
Non-anastomic biliary strictures	27 (12.6)	9 (15.3)	15 (14.6)	3 (5.8)	0.218	9/214
Ischemic type biliary lesion	15 (7.0)	3 (5.1)	11 (10.7)	1 (1.9)	0.099	
Intrahepatic biliary lesions	12 (5.6)	6 (10.2)	4 (3.9)	2 (3.8)	0.251	
PTLD	2 (0.9)	0 (0)	2 (1.9)	0 (0)	0.499	9/214

Abbreviations: CMV: cytomegalovirus; ACR: acute cellular rejection; HAT: hepatic artery thrombosis; PTLD: post-transplant lymphoproliferative disorder.

Table S7. One-year outcome and indirect effects of CMV infection related to virus load (IU/ml; *n*=50).

	**All (*n* = 50)**	***>125-500* (*n* =24)**	***501-1000* (*n* = 7)**	***>1000* (*n* = 19)**	**P-value**	**Missing (*n*/Total)**
Overall mortality	11 (22.0)	7 (29.2)	1 (14.3)	3 (15.8)	0.481	0/50
Time of death (days)	57 (15-341)	161 (29-341)	15 (-)	49 (40-220)	0.250	
Graft failure	5 (10.0)	3 (12.5)	2 (28.6)	0 (0)	0.096	0/50
Time of onset (days)	9 (1-75)	52 (2-75)	5 (1-9)	0 (0)	0.248	
Retransplantation	3 (6.0)	2 (8.3)	1 (14.3)	0 (0)	0.376	
CMV relapse	18 (36.0)	8 (33.3)	2 (28.6)	8 (42.1)	0.733	1/50
Immunological complications	8 (16.0)	5 (20.8)	1 (14.3)	2 (10.5)	0.676	1/50
ACR, early-onset	5 (10.0)	4 (16.7)	0 (0)	1 (5.3)	0.367	
ACR, late-onset	2 (4.0)	0 (0)	1 (14.3)	1 (5.3)	0.167	
Chronic rejection	0 (0)	0 (0)	0 (0)	0 (0)	-	
Antibody mediated rejection	0 (0)	0 (0)	0 (0)	0 (0)	-	
Graft-versus-host disease	1 (2.0)	1 (4.2)	0 (0)	0 (0)	1.000	
Vascular complications	7 (14.0)	5 (20.8)	2 (28.6)	0 (0)	0.051	0/50
HAT, early-onset	3 (6.0)	1 (4.2)	2 (28.6)	0 (0)	0.065	
HAT, late-onset	0 (0)	0 (0)	0 (0)	0 (0)	-	
Portal vein thrombosis	2 (4.0)	2 (8.3)	0 (0)	0 (0)	0.645	
Venous thromboembolism	0 (0)	0 (0)	0 (0)	0 (0)	-	
Arterial thrombotic disease	2 (4.0)	2 (8.3)	0 (0)	0 (0)	0.645	
Non-anastomic biliary strictures	9 (18.0)	4 (16.7)	1 (14.3)	4 (21.1)	1.000	0/50
Ischemic type biliary lesion	7 (14.0)	3 (12.5)	0 (0)	4 (21.1)	0.420	
Intrahepatic biliary lesions	2 (4.0)	1 (4.2)	1 (14.3)	0 (0)	0.448	
PTLD	2 (4.0)	2 (8.3)	0 (0)	0 (0)	0.645	0/50

Abbreviations: CMV: cytomegalovirus; ACR: acute cellular rejection; HAT: hepatic artery thrombosis; PTLD: post-transplant lymphoproliferative disorder.
